# Authentic leadership: application to women leaders

**DOI:** 10.3389/fpsyg.2015.00959

**Published:** 2015-07-15

**Authors:** Margaret M. Hopkins, Deborah A. O’Neil

**Affiliations:** ^1^Management, University of Toledo, Toledo, OHUSA; ^2^Management, Bowling Green State University, Bowling Green, OHUSA

**Keywords:** authentic leadership, women leaders, individual vs. relational leadership, gendered leadership theory, critical analysis

## Abstract

The purpose of this perspective article is to present the argument that authentic leadership is a gendered representation of leadership. We first provide a brief history of leadership theories and definitions of authentic leadership. We then critique authentic leadership and offer arguments to support the premise that authentic leadership is not gender-neutral and is especially challenging for women.

## The Evolution of Leadership Theories

Although scholars have pursued the subject of leadership throughout history, the modern roots of leadership studies began in the late nineteenth and early twentieth centuries. Since that time, there have been four primary perspectives in leadership theory – the trait, the behavioral, the contingency, and the contemporary which includes transformational and authentic leadership. These four reflect distinct theoretical approaches to the field of leadership research.

The trait approach to leadership dominated much of the research during the late nineteenth and early twentieth centuries. Trait theories became known as the Great Man approach as they examined high-achieving leaders and sought to identify the distinguishing characteristics of leaders versus non-leaders. There has been a resurgence of interest in researching the personal characteristics of leaders; and humility, charisma, integrity, and optimism are examples of more recently identified distinctive leadership traits (e.g., Dinh et al., 2014). The behavioral approach to leadership research was established during the middle to late twentieth century. Early work by Lewin and Lippit (1938) identified three distinct leadership styles of behavior: the democratic, the autocratic, and the laissez faire. The Ohio State studies (Hemphill and Coons, 1957) classified two different categories of leader behaviors, task-oriented behaviors called the initiation of structure and relationship-oriented behaviors called consideration. The third primary approach to leadership research, the contingency, came into prominence in the latter part of the last century. While prior studies had emphasized the qualities of a leader and their behaviors, contingency theories accounted for both leader and situational variables. Fiedler’s (1964) contingency model, House’s (1971) path goal theory of leadership, and Hershey and Blanchard’s (1977) situational leadership theory all take into account the situational factors impacting the leader and his choice of action. The contingency approach to leadership was an attempt to develop a more comprehensive picture of leadership, to take into account the intervening variables which might explain why behavioral effects on outcomes differ across situations.

While the trait, behavioral and contingency theories of leadership remain topics of current research, contemporary perspectives reframe leadership as a dynamic and ethical process between individuals pursuing a common goal. Burns (1978) challenged the prevailing viewpoint of leadership by advocating that a primary responsibility of leaders was to develop followers into leaders themselves. His work helped differentiate between the concepts of leader and leadership, with the former reflecting a position and the latter representing a collaborative process. Building on Burns’ work, transformational leadership (Bass, 1985; Bass and Riggio, 2006) focuses on how the leader affects followers by increasing their awareness of the importance and value of their tasks, helping them focus on collective goals and motivating them through their higher-order needs.

## Authentic Leadership: A Contemporary Perspective

Authentic leadership has its origins in Greek philosophy, humanistic psychology, and more recently in the field of positive psychology (Avolio and Gardner, 2005). The ancient Greek philosophical meaning was expressed in terms of individual values and ethical choices; and contemporary philosophers focus on authenticity as a state that helps resolve the tension between individual norms of responsibility and the collective norms of moral conformity (Novicevic et al., 2006). The humanistic psychological perspective considered the development of fully functioning or self-actualized individuals who see themselves clearly and accurately and are not hindered by others’ expectations for them (Maslow, 1971).

Popular attention was given to the topic of authentic leadership through George’s (2003) book, written in response to a series of ethical lapses on the part of corporate leaders. George noted that authentic leaders pursue purpose with passion, practice values, lead with heart and head, establish long-lasting meaningful relationships, and demonstrate self-discipline. Kernis (2003) further conceptualized authenticity as comprising the following four elements: self-awareness; unbiased processing; relational authenticity; and authentic behaviors. He proposed that authenticity is developed through self-esteem moments, where people may get in touch with their true selves or alternatively conform to social pressures and norms.

Building on this work, authentic leadership has been defined more recently as “a pattern of leader behavior that draws upon and promotes both positive psychological capacities and a positive ethical climate, to foster greater self-awareness, an internalized moral perspective, balanced processing of information, and relational transparency on the part of leaders working with followers, fostering positive self-development” (Walumbwa et al., 2008, p. 94). Self-awareness is conceptualized as an understanding of how one makes sense of the world and how this process influences one’s self-concept, strengths, and weaknesses. Internalized moral perspective is self-regulation guided by internal moral standards. Balanced processing of information is described as the objective analysis of relevant information before decision-making. Relational transparency is the presentation of one’s authentic, true self to others.

There has been increased attention to the concept of authentic leadership in the past decade. One review of the literature (Yammarino et al., 2008) identified 23 conceptual publications and four empirical publications. Just a few years later, 41 theoretical journal articles, 23 empirical journal articles, and five practitioner journal articles were found on the subject of authentic leadership (Gardner et al., 2011). Several antecedents and outcomes of authentic leadership have been identified in these nascent empirical investigations. Examples of the antecedents include leader self-knowledge and self-consistency (Peus et al., 2012), and psychological capital defined as optimism, resiliency, and hope (Jensen and Luthans, 2006). Studies have found positive relationships between authentic leadership and outcomes such as trust in leadership (Hunt et al., 2008), follower job performance moderated by follower positive psychological capital (Wang et al., 2014), leader and follower well-being (Gardner et al., 2005), satisfaction with supervisor (Walumbwa et al., 2008), organizational citizenship behaviors (Cottrill et al., 2014), and organizational commitment (Jensen and Luthans, 2006; Peus et al., 2012).

Three central themes emerge from a review of the authentic leadership literature: a focus on self-awareness, an emphasis on the true self, and a grounding in moral leadership. An important distinction between authentic leadership and other theories of leadership is the prominence of the deep sense of self on the part of the leader (Avolio and Gardner, 2005).

Critical attention has also been given to the concept of authentic leadership. First, the definitions of the construct have been criticized (Chang and Diddams, 2009; Ibarra, 2015). As currently defined, authentic leadership is a broad notion which includes a range of traits, qualities, and behaviors (Chang and Diddams, 2009). Avolio and Gardner (2005, p. 316) suggest that researchers concentrate on authentic leadership as a “root construct underlying all forms of positive leadership……” which paints a wide-ranging picture with multiple dimensions and unclear boundaries. Another concern is that the definition of authentic leadership is a utopian one. Authentic leadership theory makes the assumption that the values and purpose of the leader and his followers will be fully congruent, and that authenticity will be transmitted from the leader to the followers. Yet authenticity is not a property which can be given to another individual (Algera and Lips-Wiersma, 2012). A further utopian perspective of authentic leadership theory is the balanced processing of information, identified as one of the four core components (Kernis, 2003; Avolio and Gardner, 2005). The supposition here is that one can remain unbiased in gathering and analyzing information. A related area of criticism revolves around the potential for authentic leaders to be seen as arrogant and overly self-confident, as these leaders are presented as being superior to others who are not so authentic (Algera and Lips-Wiersma, 2012). Does the expression of the true self always result in positive outcomes? These issues lead to the question “can there be a dark side to authentic leadership?”

Ibarra (2015) questions the focus on being true to self in authentic leadership. She advocates that authentic leadership should not be seen as an unwavering sense of self but as an adaptive self, since effective leaders are constantly seeking to learn about themselves and identity is an evolving life story. Existentialists also maintain that the self is not truly authentic in everyday life (Algera and Lips-Wiersma, 2012). A further primary concern is the weight placed on the self as opposed to the self in relation with others. Authentic leadership theory proposes that one can discover one’s true self by oneself (Berkovich, 2014). This perspective assumes individual agency and that “organizational life is viewed as the result of individual action” (Hosking et al., 1995, p. 10). However, discovering oneself is both an introspective and a social process (Ibarra, 2015). Leadership occurs in relational dynamics and therefore the true self is actually the self in relation to others (e.g., Anderson and Chen, 2002). Through this relational viewpoint, self, and other are not separated but are constantly constructing the meaning and reality of leadership (Uhl-Bien, 2006). Thus, the expression of authenticity in authentic leadership is both an individual and a collective responsibility.

## Authentic Leadership: A Gendered Construct with Challenges for Women Leaders

Applying a gender-neutral framework to authentic leadership ignores the sex-related differences in leadership (e.g., Eagly and Johnson, 1990; Sharpe, 2000; Eagly et al., 2003; Ely and Rhode, 2010) and does not take into account the gendered contexts in which women work (O’Neil et al., 2008, 2015). Yet discussions of authentic leadership neglect to address the gendered nature of this contemporary leadership construct. Authentic leadership is especially challenging for women leaders for three interrelated reasons. First, there is a double-bind dilemma for women in leadership (Catalyst, 2007; Eagly and Carli, 2007). A “think manager, think male” mindset is still the predominant perspective and masculine leadership behaviors such as assertiveness and competitiveness remain the norm (Schein, 1973, 2007). Thus women are caught between impossible choices and “…are often perceived as going against the norms of leadership or those of femininity” (Catalyst, 2007, p. 1). If they are highly ambitious and self-confident (agentic behaviors typically associated with men), then women may be criticized for lacking communal qualities; and if they are highly communal (helpful or friendly, typically associated with women), then women may be criticized for not being agentic enough (Eagly and Carli, 2007). Role congruity and the lack of fit theories (Heilman, 1983; Eagly and Karau, 2002) explain this dilemma whereby the requirements of the leader role and the female gender role are often inconsistent. Given the emphasis on the true self in authentic leadership, how can women enact their true self when they are faced with this double-bind? Maslow (1971) discussed the authentic or true self as one who is not hindered by others’ expectations of them; and Kernis (2003) proposed that the true self is not developed through conforming to social norms or pressures. However, women need to stay within a “narrow band of acceptable behavior – to combine seemingly contradictory behaviors” (Morrison et al., 1992, p. 54). To illustrate this point, a recent study examined the media representations of one male CEO and one female CEO of two major Australian banks during the global financial crisis in the latter part of the past decade (Liu et al., 2015). Through a discourse analysis of the media reports on these two banking executives during this time of crisis, the male CEO was represented as a James Bond heroic change agent. On the contrary, the relational and nurturing sides of the female CEO’s leadership were highlighted. For the female CEO, the media focused on a “discourse of difference” (Liu et al., 2015, p. 249) which stressed her feminine identity. In this study, authenticity was associated with “…stereotypes of what it means to be a man (independent, strong, active and decisive) and a woman (nurturing, caring, outgoing and communal”; Liu et al., 2015, p. 249). When the male CEO acted decisively, he was seen as being authentic. When the female CEO acted decisively, she was portrayed as inauthentic. The authors concluded that the social construction of authenticity was dependent on the leaders performing authenticity aligned with the appropriate gender norms.

A second reason behind the concerns of gender-neutrality applied to authentic leadership is that organizations themselves are not gender-neutral but are gendered.

“…even the most progressive modern organizations have been created by and for men, and thus tend to have systems, policies, norms, and structures that favor the male life experience. Behaviors and values regarded as the norm at work tend to favor traits and characteristics traditionally associated with maleness and to undervalue traits and characteristics traditionally associated with femininity” (Ruderman and Ohlott, 2005, p. 4).

Scenarios that cause women to question whether they are living authentically include: when reconnecting with goals and dreams; when faced with a major life event; when a changing situation places values and behaviors in conflict; and when trying to fit in a male-dominated organizational environment (Ruderman and Ohlott, 2005). The last circumstance is a constant challenge facing women in leadership roles and thus a constant source of tension in striving for authenticity. Organizations often reward individual achievements which results in women feeling less than authentic because their leadership styles and behaviors tend toward the collaborative and relational which are undervalued (Eagly et al., 1995).

Finally, the criticism that authentic leadership places an inordinate emphasis on the self and individual agency vs. the self in relation to others is relevant to the gendered nature of authentic leadership. “The emphasis on leaders being true to themselves so that they can influence others through displays of their values and beliefs is curiously one-sided” (Eagly, 2005, p. 460). Eagly (2005) proposed that attention be given to the features of authenticity that exist in the relationships between leaders and followers, since leadership is about followers’ reactions as well as leader’s actions. The processes that occur between a leader’s enactment of values and her followers’ connection with those values is a core aspect of relational authenticity as defined by Eagly (2005). One investigation of the relationship between authentic leadership, gender, psychological capital, and positive work climate found that the predominantly male authentic leaders provided a slightly less positive climate for female than for male followers (Woolley et al., 2011). These authors proposed that the values between the male leaders and female followers may have been incongruent, or the operating definition of authentic leadership may be inherently masculine. Following this argument, Ibarra (2015) also suggests that the operating definition of authentic leadership is based on individualistic (traditionally seen as masculine) vs. collective (traditionally seen as feminine) ideals. Considering women in leadership roles, the arguments have been presented that women are not always accepted as legitimate leaders due to gender bias (e.g., role incongruity, Eagly and Karau, 2002). Thus a female leader may experience feelings of being the outsider with a greater difficulty in obtaining her followers’ trust and acceptance as an authentic leader.

## Summary

Authentic leadership is a contemporary leadership perspective which places emphasis on the leader’s understanding of his true self and his actions that align with his true self. The current literature on authentic leadership describes leaders in heroic terms, which reinforces the stereotypical individualistic agency of leadership as opposed to recognizing or rewarding the relational aspects of leadership. This viewpoint of authentic leadership also neglects to address how authentic leadership applies to women and the particular concerns facing women leaders who want to enact authentic leadership. We presented three primary issues which result in authentic leadership being particularly challenging for women. First, there is a double-bind dilemma which forces women to make a choice between acting in concert with gender-normative behaviors or with expected leadership role behaviors. Second, organizations are gendered entities which require women to fit into male-dominated environments. Third, the weight given to the individual, true authentic self as opposed to the self in relation to others continues to position women as leadership outsiders due to the focus on the traditionally masculine, individual agentic aspects of leadership. We propose that these three concerns facing women leaders should be explored and integrated into the ongoing investigations of the construct of authentic leadership. This will result in authentic leadership being a more inclusive concept and an ideal toward which all leaders can strive.

## Conflict of Interest Statement

The authors declare that the research was conducted in the absence of any commercial or financial relationships that could be construed as a potential conflict of interest.
